# Clinical significance and functional validation of PPA1 in various tumors

**DOI:** 10.1002/cam4.894

**Published:** 2016-09-26

**Authors:** Dehong Luo, Guanwen Wang, Wenzhi Shen, Shuangtao Zhao, Wei Zhou, Lin Wan, Liying Yuan, Shuang Yang, Rong Xiang

**Affiliations:** ^1^ Chongqing Key Laboratory of Molecular Oncology and EpigeneticsThe First Affiliated Hospital of Chongqing Medical UniversityChongqing400016China; ^2^ Tianjin Key Laboratory of Tumour Microenvironment and Neurovascular RegulationMedical College of Nankai UniversityTianjin300071China; ^3^ The First People's Hospital of ZunyiZunyi563002China

**Keywords:** Apoptosis, bioinformatics analysis, PPA1, proliferation, tumors

## Abstract

The aim of the study was to detect PPA1 expression in various tumors and to investigate the relationship between PPA1 expression and clinicopathological parameters to further analyze its clinical significance. Immunohistochemical staining detected PPA1 expression in 305 noncancerous tissues and 675 tumor tissues, which included 12 different tumor types. QPCR and western blot examined PPA1 expression in tumor‐derived cell lines including those derived from liver, breast, lung, and ovarian cancers. Cell proliferation and apoptosis assays were used to investigate PPA1‐regulated cell growth in tumor cells. Finally, a bioinformatics analysis was used to verify the role of PPA1 in carcinogenesis. Among the 12 types of tumors, PPA1 expression was significantly higher in lung and ovarian cancers (*P* < 0.001). In lung cancer, PPA1 expression was associated with tumor size, patients’ age, and smoking status, whereas in ovarian cancer, PPA1 expression was associated with pathological grade (*P* < 0.05). Moreover, we found that PPA1 expression was up‐regulated in lung and ovarian cancer cell lines compared with nontumor cells. In addition, suppression of PPA1 expression by RNA interference in lung and ovarian cancer cells showed increased cell apoptosis and decreased cell proliferation, which was mediated by TP53 and p21 signaling. Notably, a bioinformatics analysis was used to verify the function of PPA1 in the development and progression of tumors. PPA1 expression is significantly higher in many tumors, especially those of lung and ovarian origin, which suggests that PPA1 plays an important role in carcinogenesis and in the development of some tumors.

## Introduction

In recent decades, the incidence of malignant tumors has increased, and as a result, cancer has become a major public health problem in the United States and in many other parts of the world, which poses a serious threat to human health 1. Many researchers have focused on the early diagnosis and treatment of tumors and are continuing to explore the pathogenesis and molecular surveillance of tumors to discover new targets for the early diagnosis and clinical treatment of cancer.

PPA1 (inorganic pyrophosphatase1) exists widely in nature, as it plays essential roles in many biological processes, such as the synthesis of carbohydrates, nucleic acids, and proteins, in a variety of metabolic pathways. One such pathway involves the hydrolysis of pyrophosphate and the subsequent release of energy, which is a substitute for ATP 2. It has been reported that PPA1 is highly expressed in tumors; this is related to the increased energy of the fast‐growing tumor cells and to the regulation of cell growth and development 3, 4. In our previous work, Wang, L.N. et al. detected PPA1 overexpression in ovarian cancer by iTRAQ 5. To further understand the expression of PPA1 in other tumor types, we used PPA1 monoclonal antibodies to test PPA1 expression in 675 cases of tumor tissues, which comprised 12 different types of tumors, and 305 cases of nontumor tissues.

Here, we found that the expression of PPA1 in the 12 tumor types was different and was significantly higher in lung and ovarian cancer, and its expression in lung cancer was associated with tumor size, age, and smoking status, while in ovarian cancer, PPA1 expression was associated with the pathological grade. At the same time, we detected PPA1 expression in various cell lines, and found that PPA1 expression was higher in lung and ovarian cancer cell lines than in corresponding nontumor cell lines, which were in agreement with the immunohistochemistry results. Moreover, the silencing of PPA1 in ES2, OVCAR3, and H460 cell lines resulted in increased levels of apoptosis and the suppression of cell proliferation, which are processes that might be regulated by TP53 and p21 signaling. Finally, a bioinformatics analysis was used to verify the function of PPA1; the result was consistent with the conclusions of this study. Based on these results, PPA1 might be useful as an important predictive and prognostic factor in lung and ovarian cancers.

## Methods

### Patients and tissue samples

A total of 274 carcinoma tissue samples were obtained from patients who underwent surgical treatment at the Third Affiliated Hospital of Zunyi Medical University (TAHZMU) from January to December 2014. Informed consent was obtained from all patients. None of the patients received therapy before surgery. The tissues from all of the patients were staged according to the American Joint Committee on Cancer TNM staging system. All paraffin‐embedded tissue samples were fixed in 10% formalin and then embedded in paraffin for histologic examination. The ovarian carcinoma tissue microarray was purchased from Xi'An Alenabio Biotechnology Ltd., and the other tissue microarrays were purchased from Shanghai Outdo Bio‐tech Co., Ltd. This study was approved by the institutional ethics committees at TAHZMU and Medical College of Nankai University.

### Immunohistochemistry

Immunohistochemistry (IHC) was performed on paraffin‐embedded specimens and on human lung cancer, ovarian cancer, hepatoma, and breast cancer tissue microarrays. The samples were deparaffinized and rehydrated. Antigens were retrieved by boiling sections in citric acid buffer (pH 6.4) at 121°C for 10 min. After the endogenous peroxidase was blocked by incubating with 3% H2O2 solution in PBS for 10 min, sections were blocked with 5% goat serum and were incubated with monoclonal mouse antibodies raised against PPA1 (Lot: 12167–382, Sigma, US) at a 1:200 dilution overnight. PBS, instead of primary antibody, was used as a negative control. After extensive washing in TBST buffer (25 mmol/L Tris–HCl (pH 7.4), 150 mmol/L NaCl, 0.1% Tween‐20), sections were incubated for 60 min with HRP‐conjugated goat anti‐mouse secondary antibody (Santa Cruz Biotechnology; 1:200). The expression levels of PPA1 in the paraffin tissue and tissue microarrays were scored according to the percentage of PPA1‐positive cells and the staining intensity of each tumor specimen. Specifically, 0–25%, 26–50%, 51–75%, and 76–100% were scored as 0, 1, 2, and 3, respectively; nonsignificant brown, light brown, moderate brown, and deep brown staining intensities were scored as 1, 2, 3, and 4, respectively. The two scores were multiplied, and a score of 1–6 was considered weak, while a score of ≥6 was considered strong. The immunohistochemical evaluation was performed by two independent observers 6. The images were recorded by an Olympus BX51 Epi‐fluorescence microscope under a 20× or 40× objective (Olympus Co., Tokyo, Japan).

### Plasmid construction

PPA1 silencing sites were screened and blasted on the Invitrogen website (https://rnaidesigner. invitrogen.com/rnaiex press/index.jsp). ShRNA targeting human PPA1 and the scrambled control sequence were designed and chemically synthesized as PPA1‐shRNA1 (5′‐GGAATCAGTTGCATGA ATATTGGATCCAATATTCATGCAACTGATTCC‐3′) and PPA1–shRNA2 (5′‐GCTACTGTGGACT GGTTTATTGGATCCAATAAACCAGTCCACAGTAGC‐3′). PPA1‐sc served as the control, and the sequence was 5′‐AAAAGCTACACTATCGAGCAATTTTGGATCCAAAATTGCTCGATAGTGTAGC ‐3′. The palindromic DNA oligos were annealed to each other to form a double‐stranded oligo, which was then ligated to the linearized pLV‐H1‐EF1*α*‐puro (cat. #SORT‐B19, Biosettia Inc) vector to generate circled pLV‐EF1*α*‐shRNA‐PPA1‐Puro.

### Cell culture

Human lung cancer cell lines (H1299, H460) and human ovarian cancer cell lines (OVCAR3, ES2) were obtained from American Type Culture Collection (ATCC). All of these cancer cell lines were routinely maintained in RPMI 1640 medium supplemented with 10% fetal bovine serum (GIBCO, Grand Island, NY) and 100U/mL penicillin/streptomycin (100 × solution, Invitrogen No.15070‐63). Cell morphology and doubling times were also regularly recorded to ensure the maintenance of phenotypes. The cells were used for no more than 2 months after they were thawed. The ES2, OVCAR3, H460, and H1299 WT cells were infected with a lentivirus carrying the pLV‐H1‐shPPA1‐puro plasmid, which was followed by clonal selection in 1 *μ*g/mL puromycin to generate polyclonal cell populations with stable expression of shPPA1.

### RNA preparation and real‐time PCR analysis

Total RNA was isolated from different cell lines using TRIzol (cat. #15596‐018, Invitrogen Inc., Carlsbad, CA). Quantitative RT‐PCR was performed in a BioRad CFX96 Real‐Time PCR machine (California) using Platinum1 SYBR1 Green qPCR SuperMix‐UDG (Invitrogen), which preferentially binds to double‐stranded DNA. The specific primers for human GAPDH were as follows: 5′‐GGCATCCACGAAACTACCTT‐3′ (sense primer) and 5′‐CTCGTCATACTCCTGCTTGC‐3′ (antisense primer). The specific primers for human PPA1 were as follows: 5′‐CGCTATGTTGCGAATTTG TTC‐3′ (sense primer) and 5′‐CCAGTATGTTTATCATTGTGCC‐3′ (antisense primer). After denaturation at 95°C for 30 sec, amplification was performed at 95°C for 5 sec, 59°C for 30 sec, and 72°C for 10 sec for a total of 40–45 cycles. Fluorescence data were acquired after the extension step in the PCR reaction. Once the reactions were complete, a melting curve program was used. The 2−ΔΔCt method was used to determine the relative fold changes in mRNA. Statistical results were averaged from three independent experiments performed in triplicate.

### Western blot

Total cellular protein was prepared by lysis of the aforementioned cell lines using a radioimmunoprecipitation assay (RIPA) buffer, and the protein concentration was determined by the bicinchoninic acid (BCA) protein assay (Pierce, Rockford, IL). Protein (10 *μ*g) was loaded onto 10–12% Tris‐acrylamide gels and transferred onto polyvinylidene difluoride (PVDF) membranes (Roche, Basel, Switzerland). Blocking was performed for 60 min with 5% nonfat dry milk in TBST, and blotting was performed with primary antibodies for 12–16 h at 4°C. Primary antibodies included the following: anti‐Ki67, anti‐caspase3, anti‐P53, anti‐*β*‐actin (Santa Cruz Biotechnology Inc., Santa Cruz, CA), and anti‐PPA1 (Sigma, USA). After extensive washing with TBST, the membranes were incubated for 1.5 h at room temperature with HRP‐conjugated goat anti‐rabbit antibody or goat anti‐mouse antibody (Santa Cruz Biotechnology; 1:5000). The membrane was visualized using ECL solution (Millipore, Billerica, MA).

### 5‐ethynyl‐2′‐deoxyuridine (EdU) cell proliferation assay

In total, 1 × 10^5^ of OVCAR3‐sc and OVCAR3‐shPPA1 cells were seeded in a 24‐well plate and incubated for 24 h. Then, an EdU (5′‐ethynyl‐2′‐deoxyuridine) incorporation assay was performed to quantify cell proliferation using a Cell‐Light^™^ EdU DNA Cell Proliferation Kit (Guangzhou Ribobio Co., Ltd, Guangzhou, China) according to the manufacturer's instructions. Cell groups that were not subsequently treated with EdU served as the negative controls.

### Cell proliferation assay

Cell proliferation was quantified by the metabolic reduction of WST‐8 (CCK‐8 cell proliferation assay), which generated a growth curve at 96 h. OVCAR3‐sc and OVCAR3‐shPPA1 were seeded at a density of 2 × 10^3^ cells per well in a 96‐well plate (day 0) and incubated for an additional 4 days. At the time of collection, 10 *μ*L of the kit reagent was added to each well, and the cells were allowed to incubate for 2 h at 37°C. Thereafter, the optical density (OD) was measured at 450 nm using a 96‐well multiscanner autoreader (Thermo Electron Corp, Waltham, MA).

### Flow cytometric analysis of apoptosis

Apoptotic cells were stained with propidium iodide and Annexin V‐FITC (BD Biosciences). A flow cytometric analysis was performed in a FACS Calibur cytometer (BD Biosciences), in which a minimum of 10,000 events were recorded. Three independent assays were conducted for these experiments, and the mean values were expressed as the mean±standard deviation (S.D.).

### Functional enrichment of the network including PPA1

DAVID Bioinformatic Tod was used to complete the functional enrichment of the targeted genes. The biological processes related PPA1 were identified by conducting gene ontology analysis with this tool. In this research, *P* < 0.05 was defined a significant threshold. At last, we used Enrichment Map plugin for cytoscape to visualize the biological process organization.

### Functional analysis of PPA1

Gene expression data including 180 cancer samples from GEO datasets was to complete a coexpression network. We evaluated the relationship of each gene pair with a Pearson's correlation coefficient(r), and Fisher's asymptotic test was used to calculate the *P*‐values for the correlation coefficient of each pair. Next, the significant pair was selected according to the *P* < 0.01 and *r* > 0.8. Finally, we predicted the functions of PPA1 by the Markov cluster algorithm (MCL).

### Statistical analyses

Statistical analyses were performed using SPSS 13.0 software (Chicago, USA). The values are expressed as the mean ± S.D. Statistical analysis was performed using Student's t‐test to compare the data in GEO datasets. The significance of the differences in PPA1 expression among lung cancer, ovarian cancer, hepatoma, breast cancer, and the corresponding normal specimens was established by Fisher's exact test. The relationship between PPA1 expression and clinicopathological factors was examined by the Pearson *χ* ^2^ method. Two‐sided *P* < 0.05 was considered statistically significant.

## Results

### PPA1 is up‐regulated in human tumor tissues

**Figure 1:**
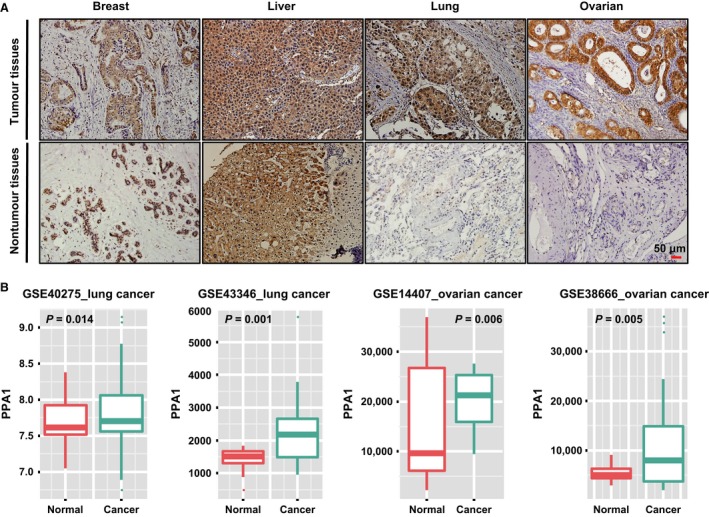
High expression of PPA1 in patients with lung and ovarian cancer (A) Immunohistochemical staining for PPA1 in paraffin‐embedded samples of human breast cancer, hepatoma, lung cancer, ovarian cancer, and their corresponding noncancer tissue samples. The expression of PPA1, which appeared as distinct brown staining located in the cytoplasm of positive cells, was recorded under a 20 ×  objective and is shown separately as representations of malignant tumors and nontumor tissue. Scale bar=50 *μ*m. (B) The PPA1 expression levels in normal tissue, primary ovarian cancer (GSE40275, GSE43346), and primary lung cancer (GSE43346, GSE38666). The expression levels are presented as boxplots and were compared using an unpaired Student's t‐test.

To examine the expression of PPA1 in tumor tissue, we first performed IHC on paraffin‐embedded carcinomas, including 12 different types of tumors, from 274 patients. As seen in Figure 1A, the immunoreactivity of PPA1 may be observed as fine, brown particles that are primarily localized in the cytoplasm and occasionally in the nuclei of cells. Our results showed that PPA1 expression in different types of tumors differed as follows: thyroid cancer (5/21 23.8%), lymphadenoma (4/21 19%), brain tumors (2/19 10.5%), and soft tissue tumors (3/12 25%) displayed weak expression, while breast cancer (15/23 65.2%), hepatoma (45/51 88.2%), lung cancer (35/44 79.5%), ovarian cancer (28/46 60.9%), bladder cancer (7/7 100%), prostate cancer (11/ 11 100%), stomach cancer (7/7 100%), and colorectal cancer (8/12 66.6%) displayed strong expression (Table 1). As the sample size of the last four cancers was too small, those cases were excluded from the follow‐up studies. Only lung cancer, ovarian cancer, breast cancer, and liver cancer were paired with corresponding normal controls and were then examined by tissue microarray to increase the sample size. The final number of specimens and the percentage of tissues that were strongly positive for PPA1 were as follows: 180 cases of breast cancer (123/180, 68.3%), 55 cases of nonbreast cancer tissue (38/55, 69.1%), 123 cases of hepatoma (87/123, 70.7%), 76 cases of normal liver (44/76, 57.9%), 191 cases of lung cancer (133/191, 69.6%), 159 cases of normal lung (7/159, 4.4%), 71 cases of ovarian cancer (47/71, 66.2%), and 15 cases of normal ovary (0/15, 0.0.%). These results indicated that PPA1 expression is significantly up‐regulated in the lung and ovarian cancerous tissues compared with their corresponding noncancerous counterparts (Table 2, P < 0.001).

**Table 1:** PPA1 expression in paraffin tissues of 12 types of carcinomas detected by immunohistochemistry

Tumor type	TC	ML	BM	STN	BTA	PCa	PGC	CRC	BC	HCC	LC	EOC
IHC++‐+++	5	4	2	3	11	7	7	8	15	45	35	28
IHC0‐+	16	17	17	9	0	0	0	4	8	6	9	18
Total	21	21	19	12	11	7	7	12	23	51	44	46
Positive rate (%)	23.8	19.0	10.5	25.0	100.0	100.0	100.0	66.6	65.2	88.2	79.5	60.9

TC, thyroid cancer; ML, malignant lymphoma; BM, brain tumor; BTA, bladder cancer; STN, soft tissue neoplasm; PCa, prostate cancer; PGC, primary gastric carcinoma; CRC, colorectal cancer; BC, breast cancer; HCC, hepatic cell carcinoma; LC, lung cancer; EOC, epithelial ovarian cancer.

Total *N* = 274.

**Table 2:** PPA1 expression in cancer and noncancerous tissues detected by IHC

	Tissue type							
	Breast cancer		Hepatocarcinoma		Lung cancer		Ovarian cancer	
Grade	Malignant	Noncancer	Malignant	Noncancer	Malignant	Noncancer	Malignant	Noncancer
IHC++‐+++	123	38	87	44	133	7	47	0
IHC0‐+	57	17	36	32	58	152	24	15
Total	180	55	123	76	191	159	71	15
Positive rate (%)	68.3	69.1	70.7	57.9	69.6	4.4	66.2	0.00

Breast cancer versus nonbreast cancer *P* = 1.000; Hepatocarcinoma versus nonhepatoma tissue *P* = 0.067; Lung cancer, ovarian cancer versus their nontumor tissues *P* < 0.001.

To investigate the clinical significance of PPA1 in ovarian cancer and lung cancer and to verify our IHC results, we compared PPA1 expression between normal tissue and primary tumor tissue using GEO datasets and bioinformatic analysis. We found that PPA1 expression was significantly increased in the primary tumors of lung cancer patients compared with normal lung tissue in GSE40275 (*P* = 0.014) and GSE43346 (*P* = 0.001). We also found that PPA1 expression was increased in the primary tumors of ovarian cancer patients compared with normal ovarian tissue in GSE43346 (*P* = 0.006) and GSE38666 (*P* = 0.005) (Fig. 1B). As the bioinformatic analysis of PPA1 supported our IHC results, we further investigated the role of PPA1 in lung and ovarian cancers via an examination of the relationship between PPA1 expression and the clinicopathologic features of each cancer type. The relationship between the expression of PPA1 and the clinicopathological variables was analyzed by the Pearson *χ* ^2^ method. In lung cancer, lesions greater than 5 cm (*n* = 61) exhibited stronger expression of PPA1 than those less than or equal to 5 cm (*n* = 124; *P* = 0.018); patients aged less than 60 years (*n* = 105) exhibited stronger expression of PPA1 than those 60 years of age and older (*n* = 77; *P* = 0.004). Smokers (*n* = 73) also exhibited stronger expression of PPA1 than nonsmokers (*n* = 112; *P* = 0.024). In ovarian cancer, tumors of histological grades II (*n* = 12) and III (*n* = 23) revealed increased PPA1 expression compared with tumors of histological grade I (*n* = 7). No significant relationship was observed between PPA1 expression and other clinicopathological factors, such as pathologic type, stage, histological grade, tumor site, presence of lymph node metastases, and gender in lung cancer (*P* > 0.05), or between subtype, age, and stage in ovarian cancer (*P* > 0.05). Briefly, PPA1 expression in lung cancer is associated with tumor size, age, and smoking status (Table 3), but not with pathology, stage, tumor grade, tumor site, lymph node metastases, or gender; in ovarian cancer, PPA1 expression is associated with pathological grade (Table 4), but not with type, age, and stage, which suggests a relationship between PPA1 overexpression and the growth and development of tumors. Taken together, our data demonstrated that PPA1 is up‐regulated in lung and ovarian tumor tissues.

**Table 3:** PPA1 expression in relation to clinicopathological and biological parameters of lung cancer patients

	Total		Weak		Strong		*P*‐value
	*n*	Percent	*n*	Percent	*n*	Percent	
Types							*P* = 0.561
SCC	93	100%	31	33.3%	62	66.7%	
Adenocarcinoma	62	100%	19	30.6%	43	69.4%	
Adenoacathoma	9	100%	1	11.1%	8	88.9%	
Others	21	100%	8	38.1%	13	61.9%	
Stage							*P* = 1.000
I+II	105	100%	34	32.4%	71	67.6%	
III+IV	80	100%	25	31.3%	55	68.8%	
Tumor grade							*P* = 0.498
I	9	100%	3	33.6%	6	66.7%	
II	134	100%	45	33.6%	89	66.4%	
III	42	100%	10	23.8%	32	76.2%	
Sites							*P* = 1.000
Left	81	100%	26	32.1%	55	67.9%	
Right	104	100%	33	31.7%	71	68.3%	
Lesion size							*P* = 0.012
>5 cm	61	100%	12	19.7%	49	80.3%	
≤5 cm	124	100%	47	37.9%	77	62.1%	
Lymph node metastases							*P* = 0.526
Yes	79	100%	23	29.1%	56	70.9%	
No	106	100%	36	34.0%	70	66.0%	
Age							*P* = 0.004
≥60	77	100%	34	44.2%	43	55.8%	
<60	108	100%	25	23.1%	83	76.9%	
Gender							*P* = 0.133
Man	144	100%	50	34.7%	94	65.3%	
Woman	41	100%	9	22.0%	32	78.0%	
Cigarette smoking							*P* = 0.024
Yes	73	100%	16	21.9%	57	78.1%	
No	112	100%	43	38.4%	69	61.6%	

**Table 4:** PPA1 expression in relation to clinicopathological and biological parameters of ovarian cancer

	Total		Weak		Strong		*P*‐value
	*n*	Percent	*n*	Percent	*n*	Percent	
Types							*P* = 0.280
Serous adenocarcinoma	27	100.0%	7	25.9%	20	74.1%	
Mucinous adenocarcinoma	10	100.0%	5	50.0%	5	50.0%	
Endometrioid carcinoma	16	100.0%	8	50.0%	8	50.0%	
Clear cell carcinoma	7	100.0%	3	42.9%	4	57.1%	
Low differentiated adenocarcinoma	11	100.0%	2	18.2%	9	81.8%	
Histological grade							*P* = 0.008
I	22	100.0%	15	68.2%	7	31.8%	
II	17	100.0%	5	29.4%	12	70.6%	
III	32	100.0%	9	28.1%	23	71.9%	
Stage							*P* = 0.086
I+II	40	100.0%	17	42.5%	23	57.5%	
III+IV	31	100.0%	7	22.6%	24	77.4%	
Age							*P* = 0.803
≥50	35	100.0%	11	31.4%	24	68.6%	
<50	36	100%	13	36.1%	23	63.9%	

### PPA1 is increased in cell lines derived from human tumors

**Figure 2:**
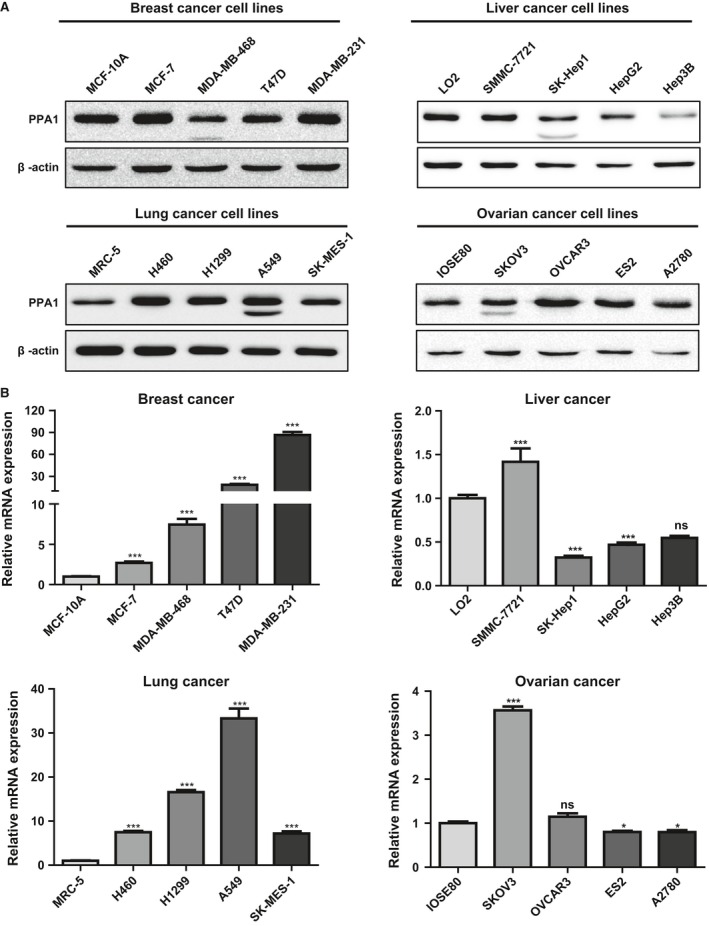
PPA1 expression in human cancer cell lines as detected by western blot and real‐time PCR. (A) The expression of PPA1 was validated by western blot analysis of four cancer cell lines and one noncancer cell line, which were derived from breast cancer, hepatoma, lung cancer, and ovarian cancer. (B) Real‐time PCR was performed to detect the expression of PPA1 in the same cell lines as in (A). *P <0.05, ***P <0.001.

After we showed that PPA1 is up‐regulated in tumor tissues, we determined whether the observations in clinical samples were also seen in tumor cell lines. A western blot analysis was conducted for the expression of PPA1 in four lung cancer cell lines (A549, H1299, H460, SK‐MES‐1), four ovarian cancer cell lines (SKOV3, OVCAR3, ES2, A2780), four hepatoma cell lines (SK‐hep1, HepG2, Hep3B, SMMC‐7721), four breast cancer cell lines (MCF‐7, MDA‐MB‐468, T47D, MDA‐MB‐231), and their corresponding noncancerous cell lines (MRC5, IOSE80, L02, MCF‐10A). The results showed that PPA1 is highly overexpressed in all of the lung cancer and ovarian cancer cell lines compared with both of their corresponding noncancerous cell lines (MRC5, IOSE80), while these same results were not obtained in the hepatoma and breast cancer cell lines (Fig. 2A). This data confirmed that PPA1 expression at the protein level was consistent with the IHC results. Additionally, this trend seen in lung cancer was consistent with the data obtained by quantitative RT‐PCR (Fig. 2B), which indicates that PPA1 expression is up‐regulated at both the mRNA and protein levels in lung cancer. The discrepancy between the protein and mRNA of ovarian cancer and breast cancer tissues might be due to differences in transcription and translation. Together, this data demonstrated that PPA1 is increased in lung and ovarian cancer cell lines.

### PPA1 deficiency fosters apoptosis in vitro

**Figure 3:**
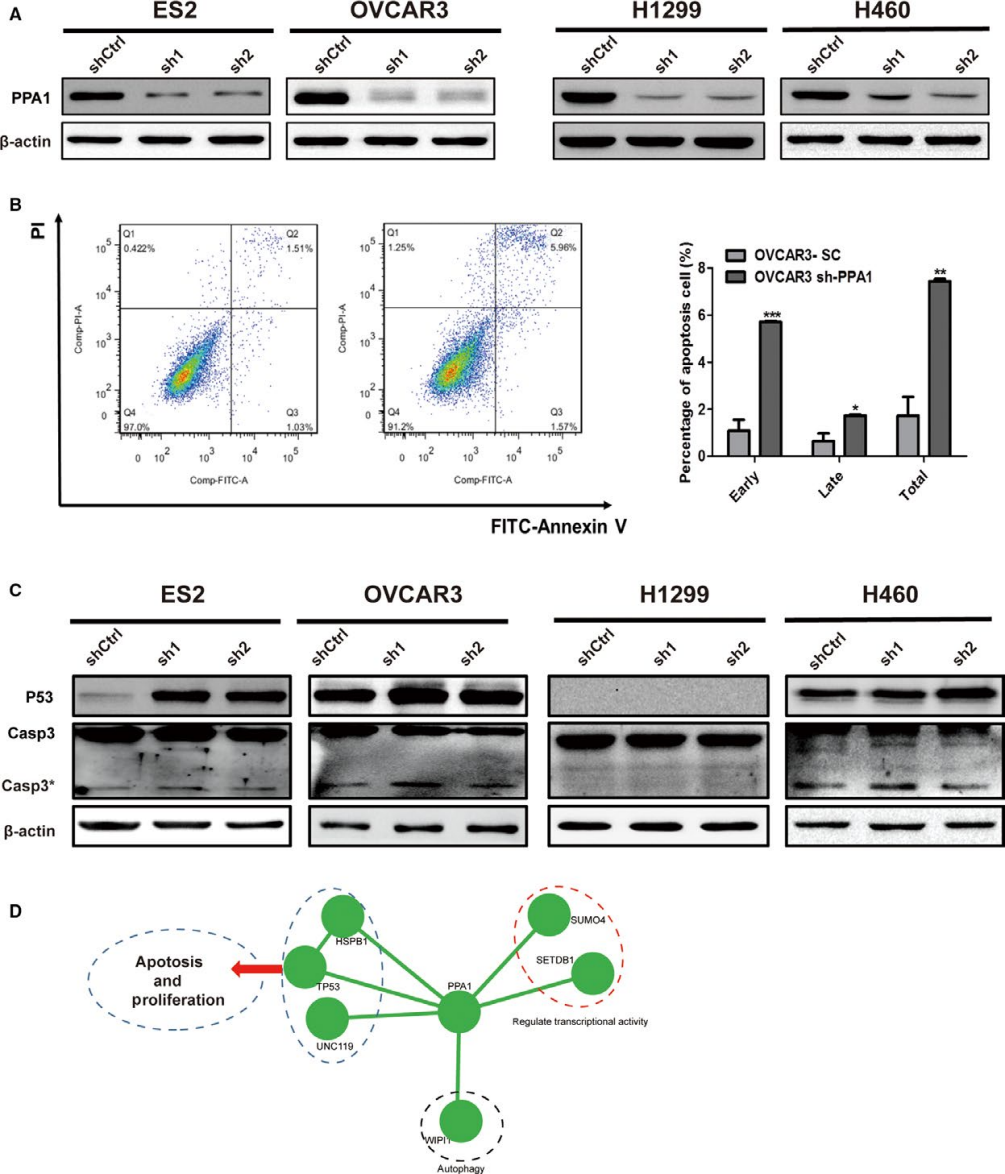
PPA1 deficiency fosters apoptosis in vitro. (A) The western blot results showed the efficiency of PPA1 knockdown in ES2, OVCAR3, H460, and H1299 PPA1‐transduced cell lines. (B) A flow cytometric analysis showed the percentage of dead cells detected by PI‐Annexin V double staining of shCtrl and shPPA1 OVCAR3 cells. A representative of three experiments are shown. The statistical results of the percentage of apoptotic cells are also shown. (C) The western blot shows the relative PPA1 expression as well as the expression of P53 and cleaved caspase‐3 in ES2, OVCAR3, H460, and H1299 cell lines with PPA1 knockdown. *β*‐actin is included as a loading control. (D) Functional enrichment maps centered on PPA1 in tumors. The gene set for GO terms were visualized using the Cytoscape Enrichment Map plugin. Each node represents a GO term. The enriched TP53 functional node around PPA1 is shown, which may indicate that PPA1 can regulate apoptosis and proliferation. *P <0.05, **P <0.01, ***P <0.001.

Our previous data showed that PPA1 expression is associated with tumor size in lung cancer, which may have resulted from apoptosis. To further investigate the function of PPA1 involvement in tumor cell apoptosis, we established ES2‐, OVCAR3‐, H460‐, and H1299‐PPA1 knockdown cell lines. The deficiency of PPA1 in these transduced cell lines was confirmed by western blot, and the results showed that PPA1 was silenced in these cell lines (Fig. 3A). To further explore the function of PPA1 in cell apoptosis, we performed PI‐Annexin V double staining and found that both early‐ and late‐stage apoptosis was increased in OVCAR3‐PPA1 knockdown cell lines compared with the control cells (Fig. 3B). At the same time, we also detected the levels of the apoptosis‐related proteins TP53 and cleaved caspase‐3, and we found that after the knockdown of PPA1, the expression of TP53 and cleaved caspase‐3 was increased significantly (Fig. 3C). This indicates that TP53 may play an important role in PPA1‐induced apoptosis. Coincidently, the cell line H1299 served as an ideal negative control since it is p53‐deficient. Moreover, no changes were observed in cleaved caspase‐3 after the knockdown of PPA1 (Fig. 3C).

Of particular note is the relationship between PPA1 and TP53, since these proteins might activate caspase and P21 activity, which may represent a key mechanism that promotes tumorigenesis. Additionally, after a network analysis that centered on PPA1, we found an enriched TP53 functional complex around PPA1, which may indicate that PPA1 can regulate apoptosis and proliferation (Fig. 3D). Accordingly, this data indicated that PPA1 deficiency promotes apoptosis in vitro.

### Silencing of PPA1 expression inhibits proliferation in vitro

**Figure 4:**
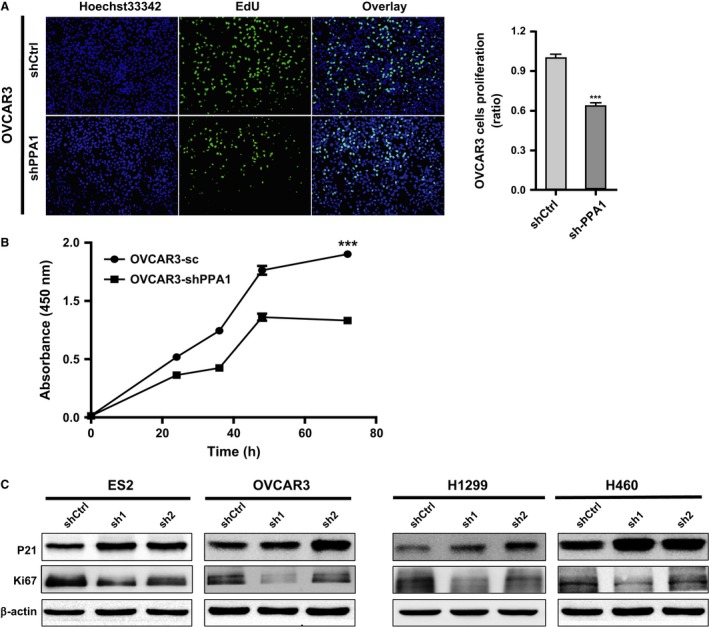
Silencing of PPA1 expression inhibits proliferation in vitro. (A) EdU incorporation assays were performed on shCtrl and shPPA1 OVCAR3 cells. (B) The cell growth of shCtrl and shPPA1 OVCAR3 cells were analyzed at 0, 24, 48, and 72 h after cell seeding. (C) The immunoblot showed the relative PPA1 expression as well as the expression of P21 and Ki67 in ES2, OVCAR3, H460, and H1299 cell lines with PPA1 knockdown. *β*‐actin is included as a loading control. ***P <0.001.

As apoptosis can inhibit cell proliferation, we also tested the function of PPA1 on cell proliferation in vitro. OVCAR3 cells in which PPA1 was knocked down were subjected to an EdU assay and showed fewer EdU‐positive cells than the control cells (Fig. 4A). We also confirmed by CCK‐8 assay that PPA1 expression led to differences in overall proliferation of OVCAR3 cells, as the results showed that OVCAR3 cells in which PPA1 was knocked down grew more slowly than the control cells (Fig. 4B). Furthermore, immunoblot results showed that the expression of the cell cycle‐related proteins P21 and TP53 was increased (Figs. 3C, 4C) and that expression of the proliferation‐related protein Ki‐67 was decreased (Fig. 4C). Collectively, these results supported the notion that PPA1 inhibits proliferation among lung and ovarian cancer.

## Discussion

According to GLOBOCAN 2012, an estimated 14.1 million new cancer cases and 8.2 million cancer‐related deaths occurred in 2012, compared with 12.7 million and 7.6 million new cancer cases and cancer‐related deaths, respectively, in 2008. The most commonly diagnosed cancers worldwide were those of the lung (1.8 million, 13.0% of the total), breast (1.7 million, 11.9%), and colon/rectum (1.4 million, 9.7%). The most common causes of cancer‐related deaths were cancers of the lung (1.6 million, 19.4% of the total), liver (0.8 million, 9.1%), and stomach (0.7 million, 8.8%). Cancer has imposed a heavy burden on society worldwide, and therefore, the discovery of novel proteins related to the genesis and progression of cancers that may affect their diagnosis, prognosis, and treatment has the potential to improve clinical strategies and outcomes for this disease. In this study, we stained 675 cases of paraffin‐embedded carcinoma tissues, 305 cases nontumor tissues, and 20 cell lines to determine whether PPA1 is up‐regulated in a variety of tumors, especially in lung cancer and ovarian cancer. We also sought to determine whether PPA1 can regulate apoptosis and proliferation through the regulation of TP53, which has been verified and evaluated by a bioinformatics analysis of the GEO database. This suggests that PPA1 plays an important role in carcinogenesis and in the development of some tumors. However, only a limited number of combined clinical and basic research reports on PPA1 has been published.

PPA1 belongs to the inorganic pyrophosphatase (PPase) family. PPases catalyze the hydrolysis of pyrophosphate (PPi) to form orthophosphate, which is vital to the phosphate metabolism of cells. It is produced as a by‐product of many of the biosynthetic reactions that utilize ATP, such as DNA and RNA synthesis 2. It is well known that PPA1 is linked to pseudogout, which is a calcium pyrophosphate dihydrate deposition disease that results when calcium pyrophosphate crystals are formed in joints, which induces pain and restricts mobility 7. Moreover, some reports on PPA1 have found that this protein mediates PKA‐induced vascular cell calcification 8, induces type I collagen expression in osteoblasts 9, and activates JNK via dephosphorylation, which regulates proliferation of mouse neuroblastomas 10 and chick cerebellar neurons 11. Nevertheless, little is known about the biological processes that involve PPA1 in the occurrence and progression of tumors 12. Some correlation has been observed between tumors and PPA1 expression. Our results indicated that PPA1 expression was significantly up‐regulated in various tumor types, especially in lung and ovarian cancer. Some reports showed that PPA1 was differentially expressed in various types of cancers, including enhanced expression in lung adenocarcinoma 4, primary colorectal cancer 3, prostate cancer 13, ovarian cancer 5, and gastric cancer 14. In addition, the up‐regulation of PPA1 in hepatic cell carcinoma (HCC) tissues showed a slight but nonsignificant trend 15. All of these reports were in accordance with our results. Furthermore, according to our IHC results, PPA1 expression in lung cancer was associated with tumor size, age, and smoking status, which conformed to the results of the study by Chen et al., who reported that PPA1 is highly expressed in patients with a positive smoking history 4. The results are also in agreement with those of Kharbhih and Panda, who found that PPA1 is highly expressed in the livers of rats and mice as a function of age 16, 17.

Furthermore, we found that PPA1 could promote cell proliferation and could protect cells from apoptosis, which may be related to the increased energy requirement of rapidly growing tumors 4, 18. TP53, which is a tumor suppressor gene that has been called the “Cellular Gatekeeper” and a “Guardian of the Genome”, is a 53‐kDa nuclear phosphoprotein that is expressed at low levels in all cells 19. Upon viral infection or DNA damage, wild‐type TP53 is up‐regulated rapidly and acts as a sequence‐specific transcription factor, which activates a large number of genes, including Bax, p21 WAF1 (p21), GADD45, and MDM2. The prime functions of TP53 involve the maintenance of a cell's genomic integrity through a dual mechanism. First, TP53 governs the G1/S phase checkpoint of the cell cycle through induction of the cyclin‐dependent kinase inhibitor p21. On the contrary, if the damage incurred is too severe, TP53 triggers apoptosis 20, 21, 22. In our study, we found that when PPA1 was silenced, the expression of TP53 increased significantly. This may trigger cell apoptosis and the inhibition of proliferation, but the specific role of TP53 in our system or whether TP53 is indispensable for cell apoptosis or proliferation, is unknown and is an area that requires further work. Additionally, an advantage of our study is that bioinformatics was applied, which confirms that PPA1 expression is significantly higher in many tumor types, especially in lung cancer and ovarian cancer, and can regulate apoptosis and proliferation through TP53. Bioinformatics is the combination of biology and information technology. This discipline encompasses any computational tools and methods used to manage, analyze, and manipulate large sets of biological data, which is an essential tool of basic and clinical research. Moreover, it is reported that HSPB1 23, 24, 25, TP53 20, 21, 22, 26, and UNC119 27, 28, 29 are associated with proliferation and apoptosis, SUMO4 30, 31 and SETDB1 32, 33 are involved in regulating transcriptional activity, more details, WIP11 34, 35 acts the role in autophagy. These genes centered on PPA1 in tumors functional enrichment maps in our network analysis, which indicates that PPA1 could make a vital effect on gene functions similar to above. And this paper mainly discusses the role of PPA1 to the proliferation and apoptosis in lung cancer, and other functions of PPA1 remains further study.

In conclusion, PPA1 expression was up‐regulated in various types of tumors compared with normal tissues, especially in lung and ovarian cancer, and was found to be involved in the increase in proliferation and the suppression of apoptosis in ovarian and lung cancer cells in vitro. Taken together, PPA1 might be useful as an important predictive and prognostic factor in patients with lung and ovarian cancers. Finally, the decreased expression of PPA1 may serve as a molecular target for the treatment of ovarian and lung cancers. However, the elucidation of the function and mechanism of PPA1 in the development of many types of cancers and its potential as a target of tumor therapy requires further exploration.

## Conflict of Interest

The authors have no conflict of interest.

## Compliance with Ethical Standards

This study was approved by the institutional ethics committees at PLAGH and Medical College of Nankai University.

## Informed Consent

Written informed consent was obtained from each individual participant.
